# Combined use of cleft and truncated triangle signs helps improve the preoperative MRI diagnosis of lateral meniscus posterior root tears in patients with ACL injuries

**DOI:** 10.1002/ksa.12597

**Published:** 2025-01-26

**Authors:** Aritoshi Yoshihara, Caroline Mouton, Renaud Siboni, Tomomasa Nakamura, Ichiro Sekiya, Hideyuki Koga, Romain Seil, Yusuke Nakagawa

**Affiliations:** ^1^ Department of Joint Surgery and Sports Medicine, Graduate School of Medical and Dental Sciences Institute of Science Tokyo Tokyo Japan; ^2^ Department of Orthopaedic Surgery Centre Hospitalier Luxembourg‐Clinique d'Eich Luxembourg Luxembourg; ^3^ Luxembourg Institute of Research in Orthopaedics Sports Medicine and Science Luxembourg Luxembourg; ^4^ Department of Orthopaedic Surgery Reims University Hospital Reims France; ^5^ Biomaterial and Inflammation in Bone Site Laboratory (EA 4691 Bios) University of Reims Champagne Ardenne Reims France; ^6^ Center for Stem Cell and Regenerative Medicine Institute of Science Tokyo Tokyo Japan; ^7^ Human Motion, Orthopaedics, Sports Medicine and Digital Methods Luxembourg Institute of Health Luxembourg Luxembourg; ^8^ Department of Cartilage Regeneration, Graduate School of Medical and Dental Sciences Institute of Science Tokyo Tokyo Japan

**Keywords:** cleft sign, ghost sign, lateral meniscus extrusion, lateral meniscus posterior root tear, truncated triangle sign

## Abstract

**Purpose:**

This study aimed to investigate whether combining the analysis of different magnetic resonance imaging (MRI) signs enhances the diagnostic accuracy of lateral meniscus posterior root tears (LMPRTs) in patients with anterior cruciate ligament (ACL) injuries. We hypothesised that analysing the cleft, ghost and truncated triangle signs and lateral meniscus extrusion (LME) measurement together would improve the preoperative MRI‐based diagnosis of LMPRTs.

**Methods:**

This retrospective study used prospectively collected registry data from two academic centres, including patients undergoing primary or revision ACL reconstruction (ACLR) and LMPRT repair. The control group included age‐ and sex‐matched (1:1) patients undergoing ACLR without any lateral meniscus tears. LME (mm) and the presence of cleft, ghost and/or truncated triangle signs were evaluated using preoperative MRI.

**Results:**

In total, 252 patients (126 per group) were included. Individually, the cleft and truncated triangle signs achieved the highest sensitivity (60% and 62%, respectively) and accuracy (>89%). The presence of either sign increased sensitivity to 79% and enabled the correct classification of 93% of ACL injuries as having or not having an LMPRT, with high specificity (95%) and good positive predictive value (74%). This combination was considered the most efficient in reducing false positives and false negatives. The LME (cutoff value: 2.2 mm) and ghost sign had lower sensitivities (50% and 14%, respectively) and accuracies (83% and 87%) and were not part of the optimal combination.

**Conclusion:**

The cleft and/or truncated triangle signs on preoperative MRI reliably detected 79% of LMPRTs in this cohort, with high specificity (95%) and good positive predictive value (74%). This combination provides an effective method for achieving reasonable sensitivity while minimising false positives, aiding surgeons in preoperative diagnosis and planning for LMPRT repair.

**Level of Evidence:**

Level III.

**Abbreviations**
ACL: anterior cruciate ligament
ACLR: anterior cruciate ligament reconstruction
AUC: area under the curve
BMI: body mass index
CI: confidence interval
IQR: interquartile range
LM: lateral meniscus
LME: lateral meniscus extrusion
LMPRT: lateral meniscus posterior root tear
MFL: meniscofemoral ligament
MME: medial meniscus extrusion
MRI: magnetic resonance imaging
NPV: negative predictive value
OA: osteoarthritis
PPV: positive predictive value
ROC: receiver operating characteristic

## INTRODUCTION

There is a general consensus that lateral meniscus posterior root tears (LMPRTs) should be systematically assessed and treated appropriately. They are known to increase anterolateral rotational knee laxity after anterior cruciate ligament (ACL) injuries [10, 22, 26, 27] and increase the risk of osteoarthritis (OA) due to the modified load distribution within the knee caused by lateral meniscus extrusion (LME) [7, 17, 20, 23, 24, 25]. However, LMPRTs have been frequently overlooked in the past although they are reported to be observed in 7%–15% of ACL injuries [1, 3, 6, 9, 21]. Their classification according to damage morphology was tentatively reported by LaPrade et al. [18] and Forkel et al. [9] and was recently updated to a new type [14, 15].

To date, no specific symptoms or physical findings associated with LMPRTs have been reported in the literature. Thus, preoperative diagnosis is, whenever possible, made using magnetic resonance imaging (MRI) or, if applicable, only possible during arthroscopy. Several characteristic signs of LMPRTs, including the cleft, ghost and truncated triangle signs, have been reported on MRI [4, 12]. LME has also been reported to be an indirect indicator of LMPRT [22]. A previous report showed that the sensitivity of these three MRI signs to establish the presence of an LMPRT was low if considered individually (34.4%–65.6%) [3]. The combination of MRI signs achieved a higher sensitivity, highlighting the potential to improve the current ability to diagnose LMPRTs before arthroscopy. However, the number of LMPRT cases in that report was relatively small, and the authors did not consider combining other indirect factors, such as LME, nor did they conclude on the best combination to consider when diagnosing LMPRT in patients with ACL injuries [3].

This study aimed to investigate whether combining the analyses of different MRI signs could improve the diagnostic accuracy of LMPRTs in patients with ACL injuries. The hypothesis was that the combined analysis of the cleft sign, ghost sign, truncated triangle sign and LME measurement would help increase the diagnostic accuracy of MRI for LMPRTs in patients with ACL injuries, thereby helping surgeons diagnose LMPRTs preoperatively and, when LMPRT is strongly suspected, prepare for its repair.

## MATERIALS AND METHODS

This retrospective study used prospectively acquired data from two ACL registries at Tokyo Medical and Dental University in Tokyo and Clinique d'Eich—Centre Hospitalier de Luxembourg in Luxembourg. All patients provided written informed consent, allowing us to use the medical data gathered during the ACL injury treatment. The study protocol was approved by the Institutional Review Board of Tokyo Medical and Dental University (M2000‐2054) in Tokyo and by the National Ethics Committee for Research (N°201101/05 version 1.0) in Luxembourg.

The present study involved the same cohort of patients from each institution as previously described [29]. Overall, 1139 patients who underwent primary or revision ACL reconstruction (ACLR) were screened (610 in Tokyo, 529 in Luxembourg). Patients were excluded if they were aged <15 years, required complex surgeries (including other ligament surgery and osteotomy), presented with a discoid lateral meniscus (LM) or had previously undergone an ipsilateral LM resection. Patients with arthroscopy‐confirmed LMPRT requiring repair and those without other concomitant LM tears were included in the LMPRT group (*n* = 126). A control group of patients without any lesions in the LM (*n* = 126) was established separately in each institution and matched to the LMPRT group according to age and sex (1:1 ratio). Previous analyses confirmed that no differences were observed between the groups in terms of sex, age at surgery, body mass index (BMI), mechanism of injury, time to MRI, time to surgery or revision ACLR [29].

### Data collection

Patient characteristics including sex, age at surgery, BMI, time from injury to MRI and time from injury to surgery (in months) were extracted.

### MRI findings

Preoperative 1.5 T or 3.0 T MRI was used to measure the LME width, as well as to assess the following MRI signs: cleft sign, ghost sign and truncated triangle sign. MRI scans were performed in the supine position with the knees slightly flexed and without axial pressure. All measurements and evaluations were performed by two trained orthopaedic surgeons (one from each institution). Both surgeons were blinded to the groups in which patients were included in the final analysis (LMPRT or control).

**Figure 1:**
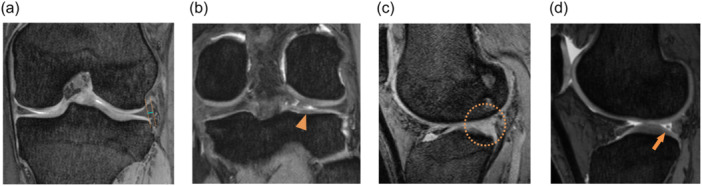
Magnetic resonance imaging (MRI) signs for a lateral meniscus posterior root tear in the left knee. (a) The lateral meniscus extrusion (LME) is evaluated on the coronal MRI slice showing the maximum extrusion of the lateral meniscus. The LME width is defined as the distance (indicated with a blue line) from the most peripheral aspect of the lateral meniscus (LM) to the border of the tibia (both indicated with orange lines). The lines are parallel to the outer margins of the femur and tibia, excluding any osteophytes. (b) On coronal images, the cleft sign is defined as a vertical linear defect around the meniscal root (indicated with an arrowhead). (c) On a sagittal slice, the ghost sign is defined as the absence of the entire contour of the LM (surrounded by a circle). (d) On a sagittal slice, the truncated triangle sign is an abrupt termination of the normal triangular meniscal contour at its tip (indicated with an arrow).

The LME width was measured on coronal slices showing the maximum extrusion of the LM. The LME width, defined as the distance from the most peripheral aspect of the meniscus to the lateral border of the tibial plateau, excluding any osteophytes, was measured in 0.1 mm increments [16] (Figure 1a). The cleft sign was defined as a vertical linear defect on the posterior root of the LM on coronal images (Figure 1b), the ghost sign as the absence of the entire contour of the LM on a sagittal slice (Figure 1c) and the truncated triangle sign as abrupt termination of the normal triangular meniscal contour at its tip on sagittal images (Figure 1d) [3, 4, 12]. All evaluations were performed within 1 cm of the LM posterior root attachment site. Each sign was reported as being absent or present [3, 22].

### Arthroscopic exploration for LMPRTs

All arthroscopies were performed by experienced orthopaedic surgeons (separately in each centre). In all cases, systematic arthroscopic exploration was performed before ACLR. Direct visualisation and probing tests for LMPRTs were performed through anteromedial and anterolateral portals with the knee held in a figure‐of‐4 position [15]. The aspiration test, which consists of activating the aspiration of a 4.5 mm shaver, was also performed [14].

### Statistical analysis

Data were analysed using MedCalc v22.030. The alpha level for statistical significance was set at *p* < 0.05 for all analyses. The sample size was based on a previously published study [29]. A power analysis using MedCalc v22.030, however, confirmed that a sample size ratio of 1/1, including 113 patients in each group, would be enough to detect a difference in the Area Under the Curve (AUC) of 0.12 to reach a statistical power of 0.8.

The Shapiro–Wilk test was used to determine whether the LME width (in millimetre) was normally distributed. When nonnormally distributed, medians and interquartile ranges (IQR; quartile 1–quartile 3) were reported, and the LME width was compared between the LMPRT and control groups using the Mann−Whitney U test. If a significant difference was observed, receiver operating characteristic curve analysis was performed to determine the optimal cutoff value of LME to distinguish between the LMPRT and control groups. The highest Youden index value was considered the optimal threshold [30]. This optimal threshold was considered for further calculations of the sensitivity, specificity and accuracy of MRI for the diagnosis of LMPRTs.

The presence or absence of these four MRI signs was evaluated in both groups. At first, AUC, sensitivity, specificity, as well as Positive/Negative Predictive value (PPV/NPV), and accuracy (percentage of correctly classified patients) were reported for each MRI sign. PPV, NPV and accuracy were corrected for an estimated prevalence of LMPRTs of approximately 15% in a population of patients with ACLR [21]. Then, the same diagnostic parameters were reported after testing different combinations of the most discriminating MRI findings. Good and very good discrimination were considered when AUC > 0.7 and AUC > 0.8, respectively.

## RESULTS

A total of 252 patients were included in the analysis (126 patients in each group).

**Figure 2:**
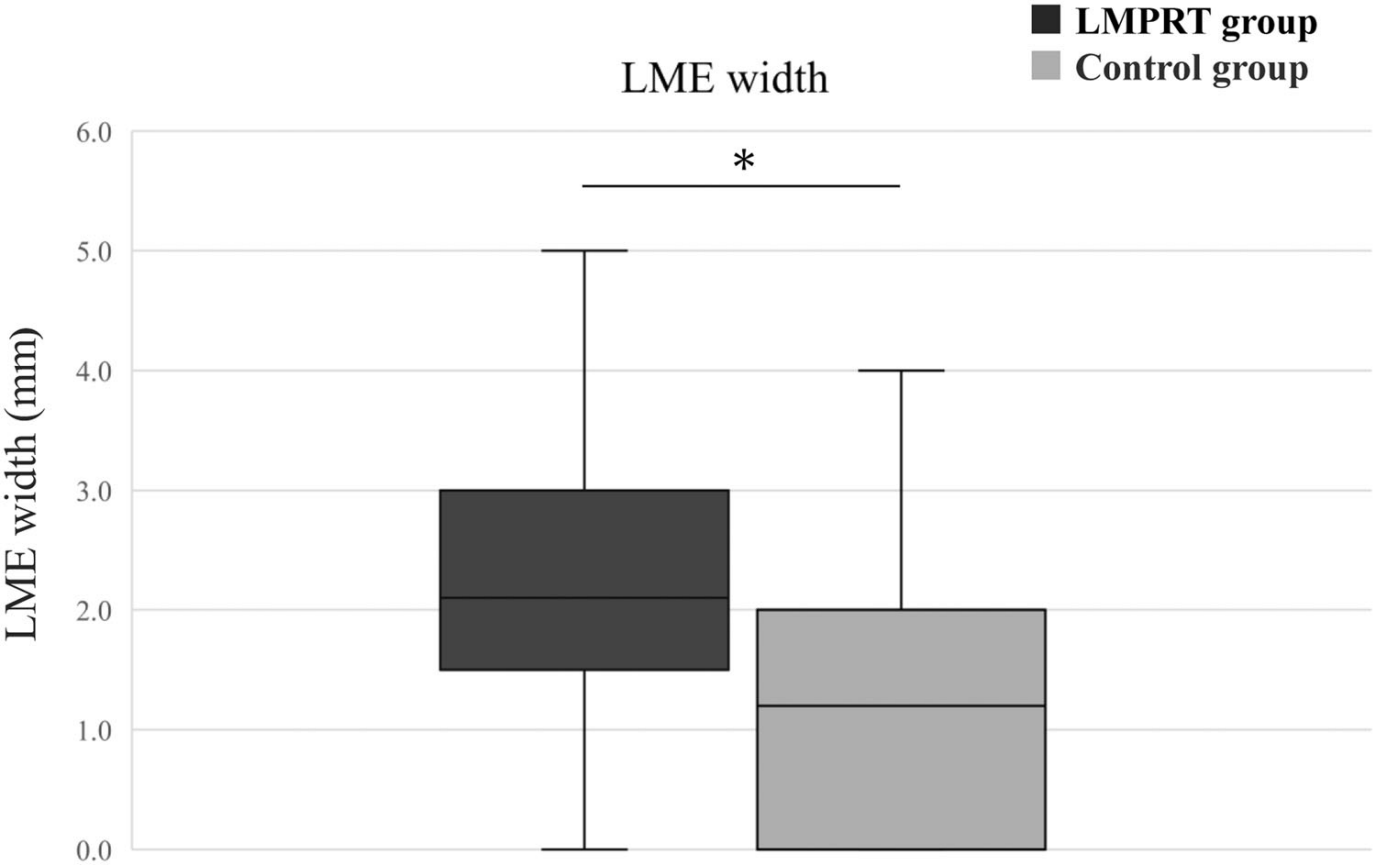
Lateral meniscus extrusion (LME) width between the lateral meniscus posterior root tear (LMPRT) and the control group. The LMPRT group displays a significantly larger LME width than the control group (*p < 0.01).

**Figure 3:**
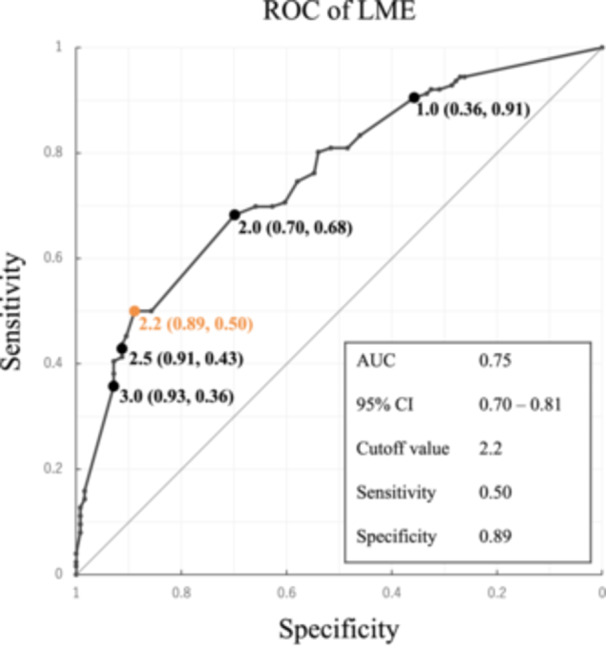
Receiver operating characteristic (ROC) curve to determine the optimal lateral meniscus extrusion (LME) width to distinguish between the lateral meniscus posterior root tear and the control group. The longest distance between the reference (diagonal) line and the curve represents the Youden Index and is obtained at a threshold of 2.2 mm. AUC, area under the curve; CI, confidence interval.

The LME width was significantly greater in the LMPRT group (median, 2.1; IQR, 1.5–3.0 mm) than in the control group (median, 1.2; IQR, 0.0–2.0 mm) (*p* < 0.01; Figure 2). The optimal cutoff value of LME width was 2.2 mm (Figure 3). This led to a sensitivity of 50% (95% confidence interval [CI]: 41%–59%), a specificity of 89% (95% CI: 82%–94%) and a percentage of correctly classified patients of 69%.

The AUC, sensitivity, specificity, PPV, NPV and accuracy corrected for the prevalence of each analysed MRI sign are shown in Table 1. Individually, the cleft and the truncated triangle signs led to the highest sensitivity (60% and 62%, respectively) and accuracy (>90%). Among these two signs, the cleft sign had a higher PPV (82%) than the truncated triangle sign (70%) owing to its higher specificity. The ghost sign showed the lowest sensitivity (14%) and AUC (0.57), indicating poor discrimination. Therefore, this was considered for further analysis. However, the ghost sign was highly specific, as it was not observed in any of the patients in the control group. This implies that when a ghost sign is observed, the presence of the LMPRT can be confirmed.

**Table 1:** Individual sensitivities and specificities of the LME width and the cleft and ghost and truncated signs for LMPRTs in patients with ACL injuries.

	AUC	*N* present in LMPRT (/126)	Sensitivity % (95% CI)	*N* absent in control (/126)	Specificity % (95% CI)	Positive predictive value^a^ % (95% CI)	Negative predictive value^a^ % (95% CI)	Accuracy^a^ % (95% CI)
LME width	0.75 (0.70–0.81)	63	50 (41–59)	112	89 (82–94)	44 (32–57)	91 (89–92)	83 (78–87)
Cleft sign	0.79 (0.73–0.83)	75	60 (50–68)	123	98 (93–100)	82 (59–93)	93 (92–94)	92 (88–95)
Ghost sign	0.57 (0.51–0.63)	18	14 (9–22)	126	100 (100–100)	100 (81–100)	87 (86–88)	87 (82–91)
Truncated triangle sign	0.79 (0.73–0.83)	78	62 (53–70)	120	95 (90–98)	70 (51–84)	93 (92–95)	90 (86–94)

Abbreviations: ACL, anterior cruciate ligament; AUC, area under the curve; CI, confidence interval; LME, lateral meniscus extrusion; LMPRT, lateral meniscus posterior root tear.

^a^ Corrected for an estimated prevalence of 15% of root tears of the lateral meniscus in a population of ACL reconstructed patients.

Table 2 reports the diagnostic parameters for different combinations of LME and the cleft and truncated triangle signs. The most accurate combination of signs was reached when considering the presence of the cleft and/or the truncated triangle sign. This combination allowed a sensitivity of 79% (17%–19% higher compared to each sign alone) and high specificity of 95%; additionally, it allowed the correct classification of 92%–93% of patients with ACL injuries as having or not having LMPRT with a good PPV (74%–80%). While other combinations also led to higher sensitivity compared to when signs were considered individually, they also displayed lower specificities and PPV. This means that other combinations of MRI signs would lead to a higher number of false positives, decreasing the PPV to 50%–61%.

**Table 2:** Sensitivities and specificities of combined LME width and the cleft and truncated signs for LMPRTs in patients with ACL injuries.

	AUC	*N* present in LMPRT (/126)	Sensitivity (%) 95% CI	*N* absent in control (/126)	Specificity (%) 95% CI	Positive predictive value^a^ % (95% CI)	Negative predictive value^a^ % (95% CI)	Accuracy^a^ % (95% CI)
LME and/or cleft sign	0.81 (0.76–0.86)	94	75 (66–82)	111	88 (81–93)	52 (40–64)	95 (94–96)	86 (91–90)
LME and/or truncated triangle sign	0.83 (0.78–0.88)	102	81 (73–87)	108	86 (78–91)	50 (39–61)	96 (95–97)	85 (80–89)
Cleft and/or truncated triangle sign	0.87 (0.82–0.91)	99	79 (70–85)	120	95 (90–98)	74 (57–86)	96 (95–97)	93 (89–96)
LME and/or cleft and/or truncated triangle sign	0.87 (0.82–0.90)	110	87 (80–93)	108	86 (78–91)	52 (41–62)	97 (96–98)	86 (81–90)
At least two out of three MRI signs present	0.79 (0.73–0.83)	75	60 (50–68)	123	98 (93–100)	81 (59–93)	93 (92–94)	92 (88–95)
All three MRI signs present	0.61 (0.56–0.68)	31	25 (17–33)	125	99 (96–100)	85 (43–98)	88 (87–89)	88 (83–92)

Abbreviations: ACL, anterior cruciate ligament; AUC, area under the curve; CI, confidence interval; LME, lateral meniscus extrusion; LMPRT, lateral meniscus posterior root tear; MRI, magnetic resonance imaging.

^a^ Corrected for an estimated prevalence of root tears of the lateral meniscus of 15%–20% in a population of ACL reconstructed patients.

## DISCUSSION

The most important finding of this study was that the presence of a cleft and/or truncated triangle sign on the preoperative MRI of a patient with ACL injury allowed for the detection of 79% of LMPRTs in this population with high specificity (95%) and PPV (74%). This combination was found to be the best among the four MRI signs studied, achieving reasonable sensitivity while avoiding a high number of false positives. It can be useful in the future to better identify LMPRTs in patients with ACL injuries, as well as to better adapt treatment, including surgical planning.

In a previous study, the combination of cleft and truncated triangle signs was reported to be more effective in the diagnosis of radial meniscal tears, with a detection rate of 76% [12]. The truncated triangle sign was initially reported as a sign of a (likely partial) radial tear [12]. Regarding LMPRTs, this sign may apply to the LaPrade classification type 2 group and oblique tears, such as type 4 [18]. In cases where a radial tear is present in the LM posterior horn, if the direction of the MRI slices is not parallel or perpendicular to the rupture site, it may not be identified as a cleft or ghost sign but may be identified as a truncated triangle sign. Furthermore, given the definitions [12], a ghost sign is diagnosed only on a single sagittal slice that is perfectly aligned with the rupture site, whereas cleft and truncated triangle signs can be potentially detected on multiple images. This could explain why the combination of a cleft and/or truncated triangle sign would be more accurate in diagnosing LMPRTs.

In the present study, the ability of LME to aid in the diagnosis of LMPRTs in patients with ACL injuries was evaluated. The optimal cutoff value of the LME width was 2.2 mm, which was almost consistent with the means of the LME width in other studies regarding LMPRT and LME, 2.0 [8] and 2.7 mm [19], respectively. Although 3 mm is a common definition of meniscal extrusion, this is the definition of medial meniscal extrusion (MME). The width of the LME is less than that of the MME in both patients with OA and healthy volunteers [11], and there is no clear definition of LME. Therefore, it was acceptable to set the cutoff value in the present study at 2.2 mm. Nonetheless, LME only helped identify 50% of patients with ACL injuries who had LMPRT. This is not substantially different from the sensitivity reported in a study that described LME as an indirect indicator of LMPRT [22], suggesting that some causes of LME without obvious tears have not yet been identified.

Similar findings could be made for the ghost sign, which displayed the lowest sensitivity (14%) in this study. However, the ghost sign was highly specific, as it was not observed in any of the patients in the control group. This implies that when a ghost sign is observed, the presence of the LMPRT can be confirmed.

Further studies should be conducted to confirm the present analyses and refine clinical recommendations. It may be interesting to further analyse whether MRI signs are related to different LMPRT subtypes according to the LaPrade or Forkel classification. Additionally, the presence of the meniscofemoral ligament (MFL) may be closely related to LME. A recent cadaveric study reported that LME was not large in cases of posterior lateral meniscal root deficiency and intact MFL, particularly when the knee was in full extension. As a result, an intact MFL may mask the presence of LMPRT and delay appropriate management [5]. Furthermore, it may be interesting to determine how the time between injury and MRI influences the presence of each MRI sign. It has been suggested that the sensitivity of each MRI sign is lower when an MRI is performed within the first 2 weeks after ACL injury [3]. Unfortunately, neither of these parameters could be assessed in the present study, as it would have required the inclusion of many more patients to obtain sufficient statistical power.

The present study has several limitations. First, patients with other LM injuries were excluded. Therefore, the specificity of the four MRI signs for LMPRT would be accurate but might be higher than actual because the fact that other types of LM injuries located in the posterior horn may induce these signs cannot be ruled out. However, the posterior horn was focused on evaluating the cleft, ghost and truncated triangle signs, which would have minimal influence. In contrast, LME results would be interpreted carefully because other LM injuries can increase LME width [2]. Second, the data were sourced from two distinct institutions where the population may differ, as well as the quality, timing and assessment of the MRI scans. However, this better represents real daily practice, where these parameters cannot always be standardised, thus enhancing the external validity of the current study. In contrast, the surgical assessment was performed by only one experienced senior knee surgeon at each institution; therefore, the authors were confident about the exhaustive identification of LMPRTs under arthroscopy (with regard to the current knowledge of these tears), thus enhancing the internal validity of the study. Therefore, we believe that the present findings and MRI signs can be easily applied in other centres and help better identify LMPRTs in patients with ACL injuries. This improvement in the identification of LMPRTs will allow better adaptation of the treatment plan, including the surgical plan, for each patient if ACLR is foreseen. Third, the method used to evaluate LME is controversial. In this study, LME width was measured as the distance from the most peripheral aspect of the LM to a tangential line drawn along the outer margins of the femur and tibia, excluding any osteophytes [16, 22]. However, an alternative method measures LME as the horizontal distance between the most external margin of the tibial plateau articular cartilage and the peripheral border of LM [28], which may yield different results. Additionally, weight‐bearing may also affect LME. Several previous ultrasound studies demonstrated that meniscus extrusion was more prominent during standing or walking compared to the supine position [13]. Moreover, load‐bearing MRI has demonstrated increased meniscal extrusion following repair [28]. Notably, all MRI scans in this study were performed with patients in the supine position. If feasible, MRI scans under weight‐bearing conditions could provide more effective and clinically relevant evaluations.

## CONCLUSION

The presence of a cleft and/or truncated triangle sign on the preoperative MRI of a patient with ACL injury allowed for the detection of 79% of LMPRTs in this population, with high specificity (95%) and PPV (74%). This combination was found to be the best option for achieving reasonable sensitivity and avoiding a large number of false positives. Accurate identification of LMPRTs on preoperative MRI can aid in operative planning and appropriate treatment.

## AUTHOR CONTRIBUTIONS

Aritoshi Yoshihara conceived the study, managed data, performed statistical analysis, participated in study design and wrote the manuscript. Caroline Mouton participated in the study design, advised statistical analysis, interpreted results and edited the manuscript. Renaud Siboni managed data, participated in study design and interpreted results. Tomomasa Nakamura, Ichiro Sekiya, Romain Seil and Hideyuki Koga participated in the study design and interpreted the results. Yusuke Nakagawa had full access to all of the data in the study and took responsibility for the integrity of the data and the accuracy of the data analysis. All authors read and approved the final manuscript and took responsibility for the integrity of the data and the accuracy of the data analysis.

## CONFLICT OF INTEREST STATEMENT

The authors declare no conflicts of interest.

## ETHICS STATEMENT

This study was approved by the Institutional Review Board in Tokyo Medical and Dental University (M2000‐2054) in Tokyo and the National Ethics Committee for Research (N°201101/05 version 1.0) in Luxembourg. All study participants provided their full written informed consent for participation in this clinical research prior to the operative procedure.

## Data Availability

Raw data were generated at Tokyo Medical and Dental University and Clinique d'Eich—Centre Hospitalier de Luxembourg. Derived data supporting the findings of this study are available from the corresponding author Y. N. on request.
